# Massive Upper Body and Cervicofacial Subcutaneous Emphysema Following Robotic Myomectomy

**DOI:** 10.1155/2019/5861705

**Published:** 2019-09-10

**Authors:** Joseph Capone, Aladino De Ranieri, Nebojsa N. Knezevic, Ivan K. Lukić, Kenneth Candido, Vicko Gluncic

**Affiliations:** ^1^Department of Anesthesiology, Advocate Illinois Masonic Medical Center, 836 W. Wellington Ave, Chicago, IL 60657, USA; ^2^Bjelovar University of Applied Sciences, Trg Eugena Kvaternika 4, Bjelovar HR-43000, Croatia

## Abstract

Subcutaneous emphysema is defined as the unintentional introduction of air or carbon dioxide in the subcutaneous tissues. The use of robotic surgical techniques has greatly expanded over the past decade specifically to treat intraperitoneal pathology. In general, advantages of these minimally invasive procedures are reported to decrease operating time, patient morbidity, and shorten hospital stay providing a safe alternative to traditional surgery. However, as with any surgery, potential complications may occur. We describe an unusual case of massive subcutaneous emphysema involving the upper body and cervicofacial region, with bilateral pneumothoraces following robotic intraperitoneal surgery. Written authorization was obtained from the patient.

## 1. Introduction

Surgical subcutaneous emphysema (SE) is a rare complication of laparoscopic and/or robotic surgery in which carbon dioxide (CO_2_) intended for abdominal insufflation spreads within the surrounding subcutaneous tissues leading to diffuse swelling and crepitus on palpation, with the potential for further extension along the fascial planes [1–5].

The overall incidence of subcutaneous emphysema following robotic assisted laparoscopic surgery is approximately 0.3%–3%. The potential risk is greater in elderly patients, with lengthy laparoscopic and robotic surgeries with more than five entry ports, and with the use of high CO_2_ insufflation pressures (15–20 mmHg) [6, 7]. The severity of SE following robotic-assisted laparoscopic surgery can be described as “mild” with crepitus at trocar insertion sites, “moderate” with crepitus extending to the abdomen and thighs, and “massive” with crepitus and swelling extending to the chest, neck, face, and extremities. Massive iatrogenic SE can potentially have life-threatening effects including hypercarbia, pneumothorax, and pneumomediastinum [1–7].

We report an unusual case of massive SE complicated with bilateral pneumothoraces related to robotic surgery with intraoperative vital signs maintained within normal limits and with the absence of a characteristic rise in end-tidal (et) CO_2_. SE particularly affected the cervicofacial region, despite the patient being in steep Trendelenburg position. Written authorization was obtained from the patient.

## 2. Case Presentation

A healthy 39-year-old female with no past medical or surgical history underwent robotic-assisted laparoscopic myectomy surgery for the presence of fibroids. Standard ASA monitors were applied, and general anesthesia was induced.

**Figure 1:**
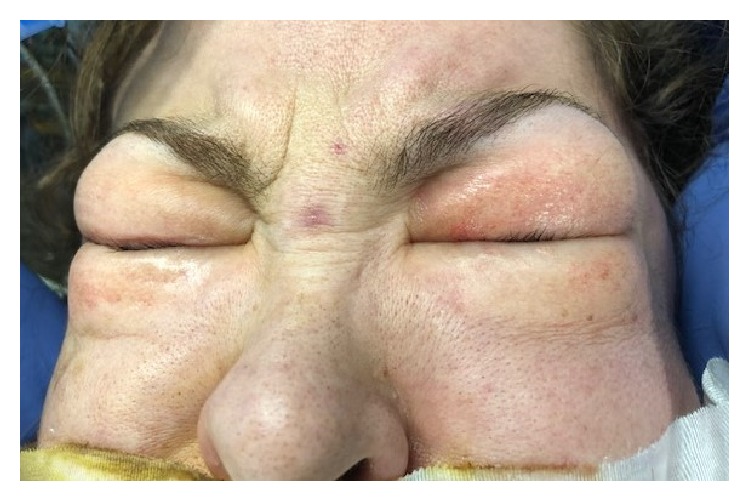
Massive subcutaneous emphysema with swelling of the face and periorbital area. The emphysema was caused by laparoscopic robotic myectomy.

**Figure 2:**
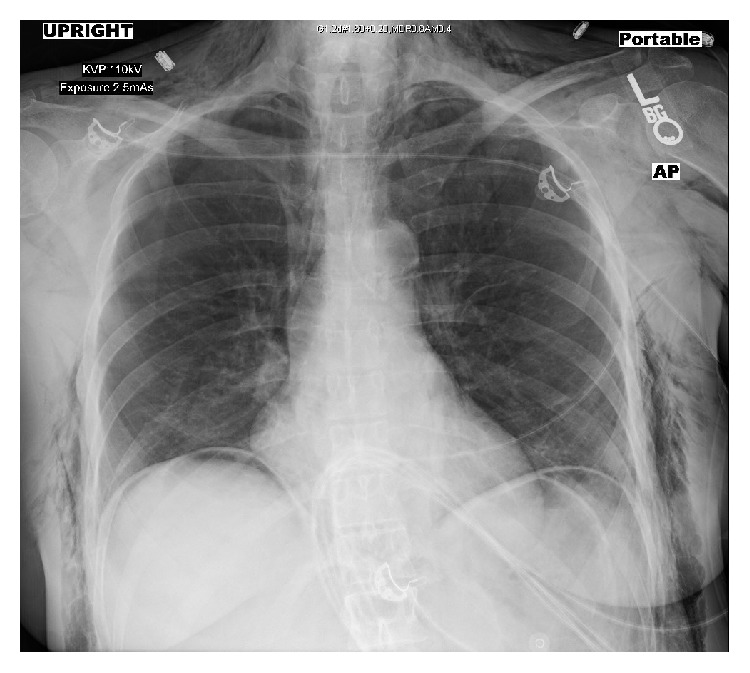
Chest X-ray showing significant subcutaneous emphysema of lower neck and bilateral chest walls, significant free intraperitoneal air, and small bilateral pneumothoraces. The emphysema was caused by laparoscopic robotic myectomy.

Pneumoperitoneum was created by CO_2_ insufflation with the intra-abdominal pressure maintained in the range of 12–15 mmHg. Intraoperatively, hemodynamic parameters, etCO_2_ and peak inspiratory pressure remained within normal limits. Maximum etCO_2_ noticed during this period was 42 mmHg and normalized by increasing minute volume of ventilation. Four hours after induction, the patient was noted to have developed marked swelling in the chest, neck, and face, particularly in the periorbital region (Figure 1). Palpation revealed skin crepitus extending from the operative site into the trunk, chest, neck, and face. Skin crepitus was particularly noticeable on the upper face involving the eyelids. The caudal extent of SE could not be appreciated due to patient positioning under the robotic surgery apparatus. Ventilatory parameters remained within normal limits at the time the SE was appreciated. The surgical team was notified and the SE was managed by lowering the peritoneal insufflation pressure, ongoing use of mechanical hyperventilation, and starting supportive measures, such as massaging the patient body, without any additional complications. At the end of the six-hour surgery, the pneumoperitoneum was deflated and upper body and facial swelling showed some instantaneous improvement. Prior to extubation, direct laryngoscopy was performed to exclude the presence of pharyngeal emphysema and laryngeal edema, which may accompany SE of the upper body leading to airway obstruction. The patient was extubated after a negative cuff-leak test ruled out airway compression by neck emphysema. A chest X-ray was taken in the post anesthesia care unit (PACU) and showed small bilateral pneumothoraces with diffuse SE (Figure 2).

The patient remained stable in the PACU and was transferred to the surgical intensive care unit (SICU) step-down unit. The patient's pneumothoraces worsened on post-operative day (POD) 1; however the patient's respiratory and hemodynamic parameters remained stable and were managed conservatively. This involved bed rest, medications to control pain, and 4 L of supplemental oxygen flow by Adult Oxygen Mask (AirLife™, CareFusion, CA, USA), which has facilitated absorption of the subcutaneous CO_2_ and/or air.

On POD 2, there was significant improvement in both the bilateral pneumothoraces as well as the SE, and the patient was transferred to a telemetry unit. In the SICU, the patient complained of severe pain throughout the whole body, which was treated by IV Dilaudid (0.2 mg IV Q15 min PRN, receiving the total of 2.4 mg over 24 h). On POD 3, the patient continued to demonstrate overall improvement in her condition, with minimal evidence of residual SE and crepitus. The patient was discharged home in stable condition with no further complications or complaints.

## 3. Discussion

Robotic laparoscopic surgery is associated with lower perioperative morbidity and mortality and is used with increasing frequency for the treatment of a variety of intra-abdominal conditions previously treated with open or standard laparoscopic surgical techniques. The main advantages of robotic surgery include a significantly shorter overall recovery time and hospital stay. There is also significantly less need for analgesia use as there is no muscle splinting. On the other hand, although the operative times of robotic laparoscopic surgeries are generally shorter, they may be longer occasionally, especially in complex cases and/or if the operator is inexperienced when compared with open surgeries—as it was in our case [1, 5]. Insufflation of the abdominal cavity with CO_2_ decreases venous return to the heart, reduces cardiac output and index, causes a marked reduction in functional residual capacity, increases peak airway pressure, increases ventilation perfusion mismatch, and leads to increased alveolar/arterial oxygen (O_2_) gradient. Insufflated CO_2_ used to create a pneumoperitoneum is absorbed from the tissues into the blood, crossing the alveolar membrane, to be expelled as CO_2_. As a result, an increase in minute ventilation of approximately 25% is needed to maintain eucarbia [3, 5, 8, 9].

One of the rare but potentially serious complications of robotic-assisted laparoscopic surgery is SE. It usually develops due to dissection around the trocar sites after repeated attempts to insert the port or at time of port removal secondary to high insufflation pressures. In these cases, CO_2_ can diffuse outside intraperitoneal and extraperitoneal cavities causing subcutaneous emphysema, pneumothorax, pneumomediastinum, pharyngeal emphysema, and/or CO_2_ embolism [1, 2, 5]. Subsequent hypercarbia may lead to dysrhythmias while PaCO_2_ above 55 mmHg can result in systolic hypertension, increased central venous pressure, tachycardia, decreased peripheral vascular resistance, and eventually leading to respiratory acidosis. In the unanesthetized patient, respiratory compensation takes place in the form of an immediate increase in ventilation and an increase in the plasma concentration of bicarbonate produced by hydration of O_2_ [1, 5, 8, 9].

The anesthetized patient, in contrast to the awake patient, is incapable of mounting a hyperventilatory response, and in the absence of adequate compensation, the serum pH can fall below 7.0 subsequently leading to cardiac dysrhythmias and depression of the central nervous system. Ensuring hypercarbia following peritoneal insufflation requires compensatory ventilatory adjustments. If etCO_2_ continues to increase, other possibilities should be ruled out. A continued rise in etCO_2_ despite the increase in the maximal voluntary ventilation (MVV) is a worrisome sign for SE and justifies temporarily ceasing insufflation until etCO_2_ returns to normal. Differences in airway pressures and increased etCO_2 _ are the earliest findings of SE. Increase of CO_2_ diffusion in SE usually causes hypercapnea and respiratory acidosis [1, 5, 8–10]. In our case, etCO_2 _ increase was absent although significant swelling of the upper body and cervicofacial region was noticeable. However, the emphysema was not detected early due to failure to examine and palpate the chest wall and neck of the patient, assuming that some degree of dependent edema is expected in surgeries of this type. Therefore, iatrogenic SE should be included in the differential diagnosis with conditions that produce increased volume of the affected areas including hematoma, allergic reaction, angioedema, or cellulitis [1, 5, 11].

Cervicofacial and periorbital SE are usually complications of massive SE in laparoscopic surgeries or complications of maxillofacial and dental procedures [5, 9, 11]. Although periorbital SE is usually benign with spontaneous resolution, it can cause ischemic optic neuritis and central retinal artery occlusion leading to visual loss. When periorbital SE shows signs of pressure effects like restricted ocular motility, sluggish pupillary reaction, disc edema or decreased visual acuity, trapped CO_2_ should be drained by needle insertion or lateral canthotomy and/or cantholysis should be performed [1, 5, 11]. For anesthesiologists, it is import to quickly identify cervicofacial SE during the surgery and to then remove or loosen tape for eye protection to alleviate pressure effect on the eye globe [11].

In most cases, there are no specific interventions for SE as it usually resolves after peritoneal desufflation. In the absence of spontaneous resolution more invasive interventions may be warranted. Those interventions include insertion of a subcutaneous catheter or microdrainage with fenestrated catheters and compressive massage. They can be combined with the elective mechanical ventilation patient until resolution of the respiratory acidosis, hypercarbia, and SE [1, 5, 10]. In our case SE persisted for 3 days after surgery with minimal presence upon hospital discharge. The spread of CO_2_ from the abdominal wall to the chest wall, extending to the face, may lead to spread of CO_2_ to pass through the mediastinum or thorax, resulting in a pseudomediastinum or pneumothorax, as it did in our case. A chest X-ray should be performed to rule out the passage of CO_2_ into the mediastinum or thorax [1, 5, 6, 10]. In addition, arterial blood gases should be drawn to evaluate the extent of the hypercarbia. Other postoperative problems include facial swelling that may temporarily impair vision and pharyngeal swelling that may cause airway compromise. An endotracheal cuff leak test should be performed prior to extubation in these cases [1, 5, 6].

The presented case illustrates the importance of frequent palpation of chest wall under the surgical drapes when the patient is having robotic-assisted laparoscopic surgery even if ventilatory parameters and etCO_2_ are within normal limits [1, 5, 10].
